# Menstrual Irregularity: A Physiological Adaptation to Cope Perceived Stress

**DOI:** 10.7759/cureus.64522

**Published:** 2024-07-14

**Authors:** Shibu S Awasthi, Sandeep Bhattacharya, Akanksha Tandon

**Affiliations:** 1 Physiology, Dr. Ram Manohar Lohia Institute of Medical Sciences, Lucknow, IND; 2 Physiology, King George's Medical University, Lucknow, IND; 3 Family Medicine, King George's Medical University, Lucknow, IND

**Keywords:** perceived stress scale-14 (pss-14), low-frequency/high-frequency ratio (lf/hf ratio), functional hypothalamic amenorrhea (fha), stress, menstrual irregularity, hrv- heart rate variability

## Abstract

Introduction: Menstrual cycle characteristics are regulated hormonally and are integrated at the level of the hypothalamus. Stress can affect the hypothalamic-pituitary gonadal axis. The objective of the study was to analyse the stress levels of women and compare their autonomic tone and menstrual characteristics.

Methodology: A group of 100 apparently healthy, young, female volunteers were included in this pilot cohort study. Subjects were assessed for perceived stress using the Perceived Stress Scale 14 Item (PSS-14) questionnaire, underwent a heart rate variability (HRV) test on the second, 10th, and 21st days of their menstrual cycle, and their menstrual history was recorded. The statistical analysis was done using Statistical Product and Service Solutions (SPSS, version 21.0; IBM SPSS Statistics for Windows, Armonk, NY) software. Metric data were expressed in terms of numerical value and analysed as mean ± SD. Paired Student’s T-test was used to compare the HRV data of all three days of the menstrual cycle separately, and p value<0.05 was considered significant. Menstrual irregularity was complained of by 13 subjects (Group A), and the rest (87 subjects) reported regular menses (Group B).

Result: The perceived stress scores of Group A were significantly higher than Group B (32.53±5.062 vs 28.057±7.618; p=0.044). On second day, Group A had higher median R-R interval (714.38±106 vs 656.84±73.50 ms; p=0.015) and lower average heart rate (85.85±12.07 vs 92.39±9.98 bpm; p=0.034) than Group B, suggesting parasympathetic dominance. On the 10th day, Group A had a higher standard deviation of heart rate (7.09±1.88 vs 5.97±1.71 bpm; p=0.032) and a very low-frequency band (1105.94±984.12 vs 730.49±557.41 μs^2^; p=0.046) than Group B, indicating parasympathetic dominance in Group A. On the 21st day, Group A had a higher standard deviation of R-R interval (58.19±20.46 vs 44.85±14.55 ms; p=0.004), root mean square standard deviation (55.71±29.84 vs 31.89±15.99 ms; p<0.001), percentage of R-R differing by 50 ms (19.20±19.58 vs 10.87±10.31%; p=0.020), total power (3,440.23±2722.29 vs 2,068.28±1,322.49 μs^2^; p=0.004), high-frequency band (1,247.57±1173.54 vs 539.06±HPO438.92 μs^2^; p<0.001), standard deviation ratio of the Poincaré plot (0.53±0.19 vs 0.39±0.16; p=0.003), normalised HF (44.0±12.9 vs 35.4±10.6; p=0.009), and a lower LF/HF ratio (1.43±0.80 vs 2.11±1.16; p=0.043) and normalised LF (53.9±14.4 vs 64.1±11.9; p=0.006) than Group B, suggesting higher parasympathetic tone of Group A than Group B.

Conclusion: Analysing these results, it can be concluded that, in apparently healthy young women, menstrual irregularity is a physiological adaptation to combat perceived stress and maintain parasympathetic dominance.

## Introduction

Menstrual cycle length and its rhythm are hormonally controlled by the functioning of the hypothalamic-pituitary-ovarian (HPO) axis being unique for every woman, normally ranging from 21 to 35 days. In the hypothalamus, the Kisspeptin-Neurokinin-Dynorphin (KNDy) neurons stimulate gonadotropin-releasing hormone (GnRH) secretion by GnRH neurons, which stimulate the anterior pituitary to secrete gonadotrophins (i.e. follicle-stimulating hormone (FSH) and luteinizing hormone (LH)) [1].

The Perceived Stress Scale 14 Item (PSS-14) questionnaire is a validated psychological tool for the assessment of perceptual stress [2]. The questionnaire comprises seven negative questions and seven positive questions about life events in the previous month, rated on a 5-point Likert scale (0-4). PSS-14 scores are obtained by reversing the responses to positive questions and then summating them with negative question responses altogether. There is no cut-off score; rather, the higher the scores, the greater the stress levels [3]. The levels of cortisol were seen elevated with higher PSS-14 scores [4].

The autonomic functioning of the body is synchronised by the sympathetic and parasympathetic nervous systems. Generally, in a healthy individual, parasympathetic dominance is seen [5]. Beat-to-beat variation of heart rate is termed as heart rate variability (HRV), a physiological phenomenon reflecting respiratory sinus arrhythmia. Other factors contributing to HRV are circadian rhythm variations, baroreceptor reflex, environmental influence, renin-angiotensin, and other humoral mechanisms. The direct determinant of HRV is the discharge rate of the sino-atrial (SA) node, influenced by sympathovagal balance. HRV is a sensitive measure of autonomic functions of the body and sympathovagal balance at any time instance. Thus, the HRV test proves to be a non-invasive electrocardiographic method for the assessment of autonomic nervous system (ANS) balance in a multitude of clinical scenarios [6].

The objective of the study was to analyse the stress levels of women and compare their autonomic tone and menstrual characteristics. The autonomic tone of the body may affect the menstrual cycle. Hence, we hypothesize that women with menstrual irregularities have altered autonomic tone of the body.

## Materials and methods

Our pilot research was a prospective cohort study. It was conducted on the premises of the Physiology Department of King George’s Medical University, Lucknow, India. Ethical clearance was obtained from the KGMU Institutional Ethics Committee (reference code: 96th ECM 11 B-thesis/p34). A total of 100 apparently healthy, young female volunteers (age group: 18-30 years) were enrolled in this study as subjects after obtaining informed consent. The volunteers were enrolled via a simple random sampling method, and they comprised young women pursuing professional courses. Confounding factors such as diet and lifestyle were matched in the cohort as a whole via detailed history and examination conducted during enrollment of the subjects. None of the subjects was involved in athletic activities. Candidates were excluded if they had any history or clinical feature suggestive of gynecological disease, any history of systemic illness, history of endocrine disorder, history of cardiovascular disease, or any history suggestive of autonomic disorder. Anthropometric data were recorded, which comprised age (in years), weight (kg), height (cm), and systolic and diastolic blood pressure (mmHg) in supine conditions. The PSS-14 questionnaire was filled out by each volunteer, and scores were evaluated and recorded. A detailed menstrual history was obtained from each subject, including cycle length and duration of menstruation. Menstrual irregularity was assessed in terms of cycle length (a parameter to express disturbed rhythm) according to the standard recommendations of the International Federation of Gynecology and Obstetrics (FIGO). Cycle length of more than 35 days was considered as menstrual irregularity [7,8]. Based on the data thus obtained, subjects were classified into Group A (suffering menstrual irregularity) and Group B (having regular menstruation).

HRV was measured in the Autonomic Laboratory of the Department of Physiology, KGMU, Lucknow, using a Lab Chart (v8.1.10; AD Instruments, Power Lab 26T, Dunedin, New Zealand). HRV was recorded as per guidelines for short-term HRV using a deep-paced breathing protocol. All the subjects were asked to take a sufficient amount of sleep, in the night prior to the test and were asked not to consume tea/coffee/nicotine or cardio-modulator substance at least six hours before the test. They were requested to wear light comfortable clothing during the HRV test and un-wear any wrist or ankle jewelry before the test itself. HRV was measured in a comfortable sitting posture on the second, 10th, and 21st days of their menstrual cycle, in afternoon timings after lunch. Median R-R, standard deviation of RR interval (SDRR), average heart rate, SD rate, root mean square standard deviation (RMSSD), pRR50, total power, VLF, LF, HF, LF/HF ratio, LFnu, HFnu, SD1, SD2, and SD1/SD2 ratio were analysed. Subjects were asked to relax for about 8-10 minutes prior to the test. During the HRV epoch hooked-up, the subject was asked to stay stable with eyes closed. ECG recording was done for five minutes at least. HRV was evaluated using time domain indices, frequency domain indices, and non-linear measurements [6] (Table 1).

**Table 1:** HRV parameters and their autonomic interpretation

Parameter		Autonomic Interpretation
Time domain parameters	Median R-R (RR interval)	Parasympathetic
	Standard deviation R-R (SDRR)	Parasympathetic [9]
	Average heart rate	Sympathetic
	Standard deviation heart rate (SD rate)	Parasympathetic
	Root mean square standard deviation (RMSSD)	Parasympathetic [9]
	Percentage R-R intervals differing by 50 ms (pRR50%)	Parasympathetic [10]
Frequency domain parameters	Total power (TP)	Sum total of frequency bands
	Very low frequency band (VLF)	Parasympathetic [11-12]
	Low frequency band (LF)	Sympathetic (majorly) [13]
	High frequency (HF)	Parasympathetic [14]
	LF/HF ratio	Sympathovagal balance [6]
	Normalised LF (LFnu)	Sympathetic
	Normalised HF (HFnu)	Parasympathetic
Non-linear parameters	Standard deviation of Poincaré plot perpendicular to the line-of-identity (SD 1)	Parasympathetic [15]
	Standard deviation of Poincaré plot in the line-of-identity (SD 2)	Sympathetic [16]
	SD1/SD2 ratio	Vago-sympathetic balance [17]

The statistical analysis was done using Statistical Product and Service Solutions (SPSS, version 21.0; IBM SPSS Statistics for Windows, Armonk, NY). Metric data were expressed in terms of numerical value and analysed as mean ± SD. As it was a pilot study, the sample size was taken as 100 subjects. A paired Student’s T-test was used to compare the HRV data of all three days of the menstrual cycle separately. p value<0.05 was considered significant.

## Results

A total of 13 subjects (Group A) reported menstrual irregularity, while 87 subjects reported regular menses (Group B), based on concurrent menstrual cycle data.

Women in both groups did not differ significantly in terms of age, height, weight, and systolic and diastolic blood pressure. They all shared the same ethnicity (South-East Asian). Group A subjects had significantly higher PSS-14 scores than Group B (32.53±5.062 vs 28.057±7.618; p=0.044) (Table 2).

**Table 2:** Comparison of demographic data and PSS-14 scores between Group A and Group B * significant (p< 0.05); PSS-14: Perceived Stress Scale 14 Item

Parameters Assessed	Group A	Group B	P-value
	(Mean ± S.D.)	(Mean ± S.D.)	
Age (years)	20.38 ± 0.836	20.18 ± 1.510	0.644
Height (cms)	158 ± 4.35	159.61 ± 6.078	0.364
Weight (kg)	55.77 ± 7.737	53.72 ± 7.426	0.340
Systolic B.P. (mmHg)	117.38 ± 7.248	115.64 ± 7.580	0.444
Diastolic B.P. (mmHg)	81.23 ± 7.297	77.56 ± 7.696	0.113
PSS-14 scores	32.53 ± 5.062	28.057 ± 7.618	0.044*

In the HRV analysis, HRV data significantly varied throughout the menstrual cycle. On the second day, Group A had a higher median R-R interval (714.38±106 ms vs 656.84±73.50 ms; p=0.015) and lower average heart rate (85.85±12.07 bpm vs 92.39±9.98 bpm; p=0.034) than Group B (Table 3), suggesting parasympathetic dominance in Group A as compared to Group B.

**Table 3:** Comparison of HRV indices on day two between Group B and Group A SDRR = standard deviation of RR interval, SD rate = standard deviation of rate, RMSSD = root mean square standard deviation, pRR50% = percentage of RR interval varying by 50 ms, VLF = very low-frequency band, LF = low-frequency band, HF = high-frequency band, SD1 = standard deviation of the Poincaré plot perpendicular to the line of identity, SD2 = standard deviation of the Poincaré plot in the line of identity, LFnu = normalised LF, HFnu = normalised HF, *significant (p<0.05)

SN	HRV Indices	Group B (n=87)		Group A (n=13)		Student's t-Test	
		Mean	SD	Mean	SD	‘t’	p-value
1-	Median RR (ms)	656.84	73.50	714.38	106.74	-2.470	0.015*
2-	SDRR (ms)	43.08	15.15	46.10	10.57	-0.691	0.491
3-	Average Rate (BPM)	92.39	9.98	85.85	12.07	2.145	0.034*
4-	SD Rate (BPM)	5.83	1.78	5.48	1.19	0.682	0.497
5-	RMSSD (ms)	33.56	21.01	35.81	17.65	-0.366	0.715
6-	pRR50%	11.36	11.99	16.94	16.70	-1.483	0.141
7-	Total power (μs^2^)	2015.51	1553.65	2244.59	959.08	-0.516	0.607
8-	VLF (μs^2^)	526.88	353.87	733.47	404.98	-1.927	0.057
9-	LF (μs^2^)	774.21	602.48	832.40	476.76	-0.333	0.740
10-	HF (μs^2^)	686.55	795.41	654.88	512.32	0.139	0.890
11-	LF/HF ratio	1.70	1.20	2.19	1.92	-1.250	0.214
12-	SD1 (ms)	23.76	14.87	25.12	12.49	-0.314	0.754
13-	SD2 (ms)	55.70	17.08	59.32	12.78	-0.734	0.465
14-	SD1/SD2 ratio	0.41	0.13	0.42	0.18	-0.283	0.778
15-	LFnu	0.569	0.141	0.589	0.191	-0.443	0.659
16-	HFnu	0.417	0.133	0.400	0.173	0.419	0.676

On the 10th day of menstrual cycle, Group A had higher SD rate (7.09±1.88 bpm vs 5.97±1.71 bpm; p=0.032) and higher VLF (1105.94±984.12 μs^2^ vs 730.49±557.41 μs^2^; p=0.046) than Group B (Table 4), this indicates that Group A subjects had relatively higher parasympathetic tone than Group B on the 10th day.

**Table 4:** Comparison of HRV indices on day 10 between Group B and Group A SDRR = standard deviation of RR interval, SD rate = standard deviation of rate, RMSSD = root mean square standard deviation, pRR50% = percentage of RR interval varying by 50 ms, VLF = very low-frequency band, LF = low-frequency band, HF = high-frequency band, SD1 = standard deviation of the Poincaré plot perpendicular to the line of identity, SD2 = standard deviation of the Poincaré plot in line of identity, LFnu = normalised LF, HFnu = normalised HF, *significant (p<0.05)

SN	HRV Indices	Group B (n=87)		Group A (n=13)		Student's t-Test	
		Mean	SD	Mean	SD	‘t’	p-value
1-	Median RR (ms)	677.72	78.28	672.23	71.97	0.238	0.812
2-	SDRR (ms)	47.25	18.20	57.14	23.15	-1.763	0.081
3-	Average Rate (BPM)	89.79	9.71	90.04	9.63	-0.085	0.933
4-	SD Rate (BPM)	5.97	1.71	7.09	1.88	-2.170	0.032*
5-	RMSSD (ms)	35.57	22.04	46.88	31.29	-1.627	0.107
6-	pRR50%	12.55	12.71	19.88	18.06	-1.828	0.071
7-	Total power (μs^2^)	2246.07	1585.56	3238.34	2306.19	-1.974	0.051
8-	VLF (μs^2^)	730.49	557.41	1105.94	984.12	-2.019	0.046*
9-	LF (μs^2^)	834.49	529.24	1074.34	661.86	-1.474	0.144
10-	HF (μs^2^)	649.77	641.54	996.90	913.07	-1.715	0.089
11-	LF/HF Ratio	1.79	1.22	1.49	0.78	0.850	0.397
12-	SD1 (ms)	25.15	15.68	33.19	22.16	-1.628	0.107
13-	SD2 (ms)	60.41	22.78	73.03	26.18	-1.827	0.071
14-	SD1/SD2 Ratio	0.41	0.13	0.42	0.15	-0.233	0.816
15-	LFnu	0.588	0.123	0.553	0.143	0.950	0.345
16-	HFnu	0.395	0.115	0.431	0.130	-1.013	0.314

On the 21st day of the menstrual cycle, Group A had higher SDRR (58.19±20.46 ms vs 44.85±14.55 ms; p=0.004), higher RMSSD (55.71±29.84 ms vs 31.89±15.99 ms; p<0.001), higher pRR50 (19.20±19.58% vs 10.87±10.31%; p=0.020), higher total power (3,440.23±2,722.29 μs^2^ vs 2,068.28±1322.49 μs^2^; p=0.004), higher HF (1,247.57±1,173.54 μs^2^ vs 539.06±438.92 μs^2^; p<0.001), higher SD1/SD2 ratio (0.53±0.19 vs 0.39±0.16; p=0.003), higher HFnu (0.440±0.129 vs 0.354±0.106; p=0.009), lower LF/HF ratio (1.43±0.80 vs 2.11±1.16; p=0.043), and lower LFnu (0.539±0.144 vs 0.641±0.119; p=0.006) than Group B (Table 5). These results signify that Group A had significantly higher parasympathetic tone and a relatively low sympathetic drive on the 21st day of the menstrual cycle than Group B.

**Table 5:** Comparison of HRV indices on day 21 between Group B and Group A SDRR = standard deviation of RR interval, SD rate = standard deviation of rate, RMSSD = root mean square standard deviation, pRR50% = percentage of RR interval varying by 50 ms, VLF = very-low frequency band, LF = low-frequency band, HF = high-frequency band, SD1 = standard deviation of the Poincaré plot perpendicular to the line of identity, SD2 = standard deviation of the Poincaré plot in the line of identity, LFnu = normalised LF, HFnu = normalised HF, *significant (p<0.05)

SN	HRV Indices	Group B (n=87)		Group A (n=13)		Student's t-Test	
		Mean	SD	Mean	SD	‘t’	p-value
1-	Median RR (ms)	663.07	87.91	689.42	75.95	-1.024	0.308
2-	SDRR (ms)	44.85	14.55	58.19	20.46	-2.915	0.004*
3-	Average Rate (BPM)	91.95	11.79	88.14	9.18	1.116	0.267
4-	SD Rate (BPM)	6.04	1.99	6.67	1.75	-1.087	0.280
5-	RMSSD (ms)	31.89	15.99	55.71	29.84	-4.387	<0.001*
6-	pRR50%	10.87	10.31	19.20	19.58	-2.365	0.020*
7-	Total power (μs^2^)	2068.28	1322.49	3440.23	2722.29	-2.952	0.004*
8-	VLF (μs^2^)	630.35	524.61	914.19	1008.29	-1.578	0.118
9-	LF (μs^2^)	889.20	601.30	1195.17	751.74	-1.655	0.101
10-	HF (μs^2^)	539.06	438.92	1247.57	1173.54	-4.100	<0.001*
11-	LF/HF Ratio	2.11	1.16	1.43	0.80	2.053	0.043*
12-	SD1 (ms)	27.79	48.72	39.44	21.13	-0.847	0.399
13-	SD2 (ms)	65.80	68.61	71.36	22.92	-0.288	0.774
14-	SD1/SD2 Ratio	0.39	0.16	0.53	0.19	-3.010	0.003*
15-	LF (nu)	0.641	0.119	0.539	0.144	2.809	0.006*
16-	HF (nu)	0.354	0.106	0.440	0.129	-2.659	0.009*

## Discussion

Menstrual irregularity was prevalent in 13% of the sample population. The PSS-14 scores of Group A being higher than Group B suggest that women with irregular menses were chronically exposed to higher levels of perceived stress. HRV analysis of Group A suggested that women with menstrual irregularity had a lower sympathetic drive and a higher parasympathetic tone as compared to women having normal menstrual rhythm.

As our study was a pilot study in this field, there remains a paucity of peer research. According to available studies conducted by Ashwini et al., Yazar et al., Schmalenberger et al., and Vishrutha et al. [18-23], it is suggested that, physiologically, there is sympathetic dominance and parasympathetic withdrawal (lower HRV) in luteal phase (represented by the 21st day of menstrual cycle) as compared to other phases, reason proposed being secretion of progesterone in luteal phase specific to ovulatory menstrual cycles. These findings were in coherence with the findings of Group B women who experienced regular menstruation. However, these findings were in opposition to the findings of Group A women who experienced menstrual irregularities.

A plausible reason for the prominent parasympathetic dominance seen on the 21st day in Group A is due to the lack of physiological progesterone surge, which is unique to ovulatory cycles. In contrast to this, Group B had a sympathetic dominance, which is generally associated with normal progesterone secretion [21]. This indicates an anovulatory cycle in Group A and a normal ovulatory cycle in Group B.

This also explains parasympathetic dominance during menses, as anovulatory cycles usually end up in painless menstruation as suggested by Bernardi et al. [24]. This occurs because of the absence of progesterone withdrawal, which induces prostaglandin secretion and normal menstrual pain. The parasympathetic tone was seen better on the 10th day in Group A because of the low level of sex steroids, which is a common feature of anovulatory cycles, as suggested by Hambridge et al. [25].

It is known from the PSS-14 score analysis that Group A subjects were under persistent stress, which is known to physiologically reduce GnRH secretion [26]. The explanation for dysregulated hypothalamic-pituitary-gonadal axis in Group A women can be attributed to functional hypothalamic amenorrhea (FHA), which is in coherence to the findings of Gordon et al., Berga, Crowley et al., and Prokai et al. [27-30]. Hence, FHA would have led to the absence of LH surge and ovulation, which results in the lowering of sympathetic drive and establishing parasympathetic dominance again.

The major limitation of the study is that, being a pilot study, the sample size was relatively small, and further in-depth research is required in this field. The study has not undertaken any hormonal profile such as cortisol, serum estradiol, serum progesterone, or ultrasonography, so the assumption of anovulation in women with irregular cycles is based on the findings of HRV analysis of previous studies, which correlate the luteal/post-ovulatory phase with sympathetic dominance.

The potential bias of the study is that it included educated women of average socio-economic status; however, other biases were ruled out by a simple random sampling method.

This pilot study conducted is one of its kind, and in the near future, 21st-day HRV may emerge as a relevant non-invasive indicator for ovulation; however, further research is warranted for quantifying normal variation in 21st-day HRV parameters over a larger population.

## Conclusions

Higher levels of perceived stress can induce menstrual irregularities in apparently healthy young women. Irregular menstrual cycle in these women is most conclusively a result of anovulation. The anovulatory cycles in young women show a typical feature of parasympathetic dominance throughout the cycle span, in contrast to ovulatory menstrual cycles, which show relative sympathetic dominance on the 21st day. It can be concluded that menstrual irregularity is a physiological mechanism of the body to cope with perceived stress.
